# A cup of coffee, what else?

**DOI:** 10.1192/j.eurpsy.2021.629

**Published:** 2021-08-13

**Authors:** P. García Vázquez, R. Gomez Martinez

**Affiliations:** Psiquiatría, Complejo Asistencial Universitario León, León, Spain

**Keywords:** caffeine, dual patology, Addiction

## Abstract

**Introduction:**

Caffeine is the worldwide most frequently consumed psychostimulant. Its availability is nearly unlimited and in Europe it is not subject to state regulation. n the DSM-5 “caffeine use disorder” is categorized as a possible future disorder that currently needs further study.

**Objectives:**

To describe clinical evaluation, diagnosis, treatment and evolution of a 24 years old female patient.

**Methods:**

A 24-year-old woman admitted to the Dual Pathology Unit with a diagnosis of: unspecified psychotic disorder, mild intellectual disability and borderline disorder. In week 17 of admission, she decided to suspend the medication, with significant improvement. Therapeutic permits increase and Wais-III is repeated, resulting in having a limited intellectual capacity. Two months after being discharged, she was readmitted with manic symptoms. The nursing staff discover that she was drinkiing a large amount of caffeine (up to 4 liters / day). After gradually stopping caffeine intake, she was discharged without psychopharmacological treatment, being able to lead a normalized life, even studying a medium degree. No more incomes were need.

**Results:**

Caffeine produces psychomotor-activating, reinforcing, and arousing effects.

**Conclusions:**

The pattern of caffeine use of patients should be considered in the medical practice. The psychostimulant properties of caffeine are reviewed and compared with those of prototypical psychostimulants able to cause substance use disorders.

